# How do respondents of primary care surveys compare to typical users of primary care? A comparison of two surveys

**DOI:** 10.1186/s12875-023-02029-1

**Published:** 2023-03-24

**Authors:** Shawna Cronin, Allanah Li, Yu Qing Bai, Mehdi Ammi, William Hogg, Sabrina T. Wong, Walter P. Wodchis

**Affiliations:** 1 Institute of Health Policy, Management and Evaluation, University of Toronto, 155 College St, Unit 425, Toronto, Canada; 2 Department of Family & Community Medicine, University of Toronto, Toronto, Canada; 3 ICES, Toronto, Canada; 4 School of Public Policy & Administration, Carleton University, Ottawa, Canada; 5 Department of Family Medicine, University of Ottawa, Ottawa, Canada; 6 Institut du Savoir Montfort, Ottawa, Canada; 7 School of Nursing, University of British Columbia, Vancouver, Canada; 8 Centre for Health Services and Policy Research, University of British Columbia, Vancouver, Canada; 9 Institute for Better Health, Trillium Health Partners, Mississauga, ON Canada

**Keywords:** Primary health care, Survey research, Nonresponse bias

## Abstract

**Background:**

Primary care surveys are a key source of evaluative data; understanding how survey respondents compare to the intended population is important to understand results in context. The objective of this study was to examine the physician and patient representativeness of two primary care surveys (TRANSFORMATION and QUALICOPC) that each used different sampling and recruitment techniques.

**Methods:**

We linked the physician and patient participants of the two surveys to health administrative databases. Patients were compared to other patients visiting the practice on the same day and other randomly selected dates using sociodemographic data, chronic disease diagnosis, and health system utilization. Physicians were compared to other physicians in the same practice, and other physicians in the intended geographic area using sociodemographic and practice characteristics.

**Results:**

Physician respondents of the TRANSFORMATION survey included more males compared to their practice groups, but not to other physicians in the area. TRANSFORMATION physicians cared for a larger roster of patients than other physicians in the area. Patient respondents of the QUALICOPC survey did not have meaningful differences from other patients who visit the practice. Patient respondents of the TRANSFORMATION survey resided in more rural areas, had less chronic disease, and had lower use of health services than other patients visiting the practice.

**Conclusion:**

Differences in survey recruitment methods at the physician and patient level may help to explain some of the differences in representativeness. When conducting primary care surveys, investigators should consider diverse methods of ensuring representativeness to limit the potential for nonresponse bias.

**Supplementary Information:**

The online version contains supplementary material available at 10.1186/s12875-023-02029-1.

## Background

The introduction of health system reforms in primary care has emphasized a need to evaluate primary care performance and patient experience. In addition to data from chart audits and administrative records, patient and physician surveys provide important information about patient and provider experience. However, recruiting primary care practices, physicians and patients to respond to surveys can be challenging [1, 2], resulting in low survey response rates [1, 3, 4]. The extent to which the survey sample represents the intended population, or survey representativeness, can help to understand the potential for nonresponse bias, that is, a failure to accurately represent the intended larger population [5].

Sampling and recruitment approaches can influence the rate and representativeness of responses [6]. One study compared participants who responded to a survey administered in a waiting room to participants who were emailed the survey, and identified differences in sociodemographic characteristics of respondents [6]. Another study conducted in Ontario emailed a survey to participants in addition to recruiting from the clinic waiting room and found that those who were consecutively sampled from the waiting room were younger and had been patients of the clinic for less time than those who responded to the email survey [7].

Representativeness can be assessed by comparing survey respondents to population data or by comparing late responders to early responders with the assumption that those who respond late are similar to those who do not respond at all [5, 8, 9]. A previous study examined the representativeness of physician and patient participants of an Ontario primary care survey, the Quality and Costs of Primary Care (QUALICOPC). Physician respondents differed slightly from their practice groups, and to a larger extent from other Ontario primary care physicians. Further, patient participants were older, had higher morbidity, and tended to be higher users of the health care system than the general population. One conclusion of this work was that visit-based sampling may have contributed to a biased patient respondent sample [4].

The sampling methods and implementation of surveys may depend on the sampling frame, budget, and setting. Broader or more targeted approaches may be used to recruit physicians, while patients may be recruited from clinic waiting rooms by practice staff, or via email. Two large-scale primary care surveys were conducted in Canada, the QUALICOPC and TRANSFORMATION surveys. We used these to compare the representativeness of physician and patient respondents attained by each approach.

## Methods

This study linked physician and patient participant data from the TRANSFORMATION and QUALICOPC surveys to Ontario population based administrative databases held at ICES. ICES is an independent, non-profit research institute whose legal status under Ontario’s health information privacy law allows it to collect and analyze health care and demographic data, without informed consent, housing administrative databases that can be used for health system evaluation and improvement. Both surveys received ethical approval, the QUALICOPC study obtained approval from the University of Toronto Research Ethics Board, and the TRANSFORMATION study received ethical approval from the Ottawa Health Science Network and Bruyère Continuing Care. All participants provided informed consent to participate in the surveys, and for the linkage of survey information and OHIP numbers to ICES. All methods were performed in accordance with relevant guidelines and regulations.

### Primary care in Ontario

Ontario is Canada’s largest province by population, with a population of 14.3 million in 2018 [10] and a universal, publicly funded health care system which covers physician primary care services. During the past 15 years, primary care has shifted from primarily fee-for-service (FFS) to 30% patient coverage by physicians in a variety of primary care models [11]. Details about primary care models are outlined in the supplementary table.

### QUALICOPC and TRANSFORMATION surveys

The QUALICOPC study was an international survey which aimed to evaluate the performance of primary care systems and meet the need for benchmarking of performance information [12]. In addition to 34 other countries, all 10 Canadian provinces participated in the study which included four surveys: a practice survey, services offered, patient values, and patient experience. In the Ontario arm of the QUALICOPC study, physicians were mailed or emailed invitations through the Ontario College of Family Physicians, to which physicians responded by enrolling on a webpage hosted by the research team. Membership in the Ontario College of Family Physicians is not compulsory for practice, but the college has a membership of over 15,000 physicians and includes most family physicians in Ontario [13]. Practice physicians were asked to collect surveys from ten consecutive consenting patients on a random date (subject to practice considerations) in the subsequent two weeks. Patients were recruited by practice staff. The full complement of surveys was completed in a single day by the majority of practices; exact patient response rates are not available. The Ontario QUALICOPC survey was administered in 2013 and early 2014 [12] and the practice response rate was 3% [4, 12].

The TRANSFORMATION study was a cross-sectional survey which aimed to improve the science of measuring and reporting of primary health care performance. It was conducted in three Canadian regions; the Eastern Ontario Health Unit was the region studied in Ontario and is the focus of this study [14]. A list of practices and physicians created from the College of Family Physicians of Canada and the local health authority was reviewed by a stakeholder advisory group and then used to recruit physician practices. Once practices agreed to participate the research team recruited a consecutive sample of a minimum of 20 patients per practice from the clinic waiting room. Data were collected between 2014 and 2016 [3]. The TRANSFORMATION survey had a practice participation rate of 41.3% and a patient participation rate of 85.5% [3]. See Table 1 for a comparison of the QUALICOPC and TRANSFORMATION surveys.

**Table 1:** Comparing the QUALICOPC and TRANSFORMATION surveys

	Quality and Costs of Primary Care	TRANSFORMATION
**Years of data collection**	2013–2014	2014–2016
**Geographic area**	Ontario 9,758 physicians	Eastern Ontario Health Unit 219 physicians
**Questionnaires included**	Practice setting survey, services provided, patient experience, patient values. All paper surveys.	Organizational survey, provider survey, Team Climate survey, patient experience. Patient survey was paper, all others were administered using software.
**Practice and physician recruitment**	Mail or email invitations sent to physicians on college list by the colleges, researchers, or sponsor organizations. One physician per practice was eligible to participate. No practice were excluded.	Letter or email sent by local decision makers, phone follow-up 10 days later. Four physician recruiters were used. Up to five physicians per practice were eligible to participate. No physicians were excluded.
**Practice and physician compensation**	Participating physicians compensated $200.	Completion honorarium for practice: $500
**Patient recruitment**	Consecutive sample of 10 patients, distributed by practice staff.	Consecutive sample of 20 patients per practice, practice staff introduced to survey administrator, who assessed eligibility and distributed surveys in waiting room.
**Patient compensation**	None	None
**Total physicians recruited**	185	26
**Total patients recruited**	1980	539
**Response rate**	Physician response rate: 3% Patient response rate: unknown	Practice participation rate: 41.3% Patient participation rate: 85.5%

Sources: [3, 4]

### Measures and data sources

Patient participants from the TRANSFORMATION and QUALICOPC surveys were linked to health administrative databases at ICES using health card numbers and physician participants were linked using unique billing numbers, which were collected as part of the survey and encrypted upon transfer. We used the Client Agency Program Enrolment (CAPE) and Ontario Health Insurance Plan (OHIP) datasets to identify patients of primary care and link them to their corresponding physicians’ identifiers. The Registered Persons Database was used to identify demographic variables, including age, sex, and level of rurality. We measured rurality using the Rurality Index of Ontario which assigns a score between 0 and 100 based on postal code [15], with 0–10 indicating an urban setting, 10 to 40 indicating small urban, and 40 or higher indicating rural. To measure for differences in socioeconomic status we used the material deprivation score from the Ontario Marginalization Index [16]. This score uses census data including the percentage of lone parent families, education level, percentage of those below the low-income measure, and percentage of houses in fair or poor condition, to assign quintiles to each census Dissemination Area (approximately 700 persons) [17], where a quintile value of 5 reflects the greatest magnitude of deprivation [18].

Patient health utilization variables for the year prior to the date of survey administration were sourced from the Discharge Abstract Database for hospitalizations, the National Ambulatory Care Reporting System for emergency department visits, and OHIP billing database for primary care visits. Resource Utilization Bands (RUB) are part of the Johns Hopkins ACG system [19]; they are a ranking system of overall morbidity from 0 to 5 based on epidemiological patterns. We used ICES derived datasets to identify participants with the following chronic disease diagnoses, based on validated case definitions: asthma [20], congestive heart failure [21], chronic obstructive pulmonary disease [22], hypertension [23], and diabetes [24]. .

Physician variables including years in practice, age, sex, and whether they graduated from a Canadian medical school, were derived from ICES Physician Database. CAPE and OHIP databases were used to identify the size of the physicians’ roster, and the primary care model type. These datasets were linked using unique encoded identifiers.

### Comparison groups

To compare physician respondents of the TRANSFORMATION survey, we created a group of physicians who worked in the same practices as the TRANSFORMATION survey physicians but did not participate in the study. The survey focused on a public health unit in Eastern Ontario, thus, the second physician comparison group was family physicians working within this geographical area who did not participate in the original study; this third group included those who were in the same practice as the survey respondents. QUALICOPC physicians were similarly compared to others from their practice groups and other primary care physicians in Ontario [4]. To compare QUALICOPC and TRANSFORMATION patient participants to other patients who use primary care we created comparison groups for each survey. We created a group of other patients who saw the responding physician on the day of survey administration. We also created a group of patients who saw each responding physician on four randomly selected dates throughout the year (one date selected in each quarter). The dates were randomly selected using analytic software, excluding the use of weekends, holidays, and days where the physician saw less than 10 patients. The use of randomly selected days was used to account for potential effects of seasonality in terms of volume and type of patients visiting the practice [25].

### Statistical analyses

We used standardized differences to examine differences between physician and patient groups because they quantify the magnitude of difference without relying on sample size [26]. Standardized differences are the difference in means of a variable between two groups divided by its standard deviation, and are also used to compare the prevalence of dichotomous variables between two groups [27]. For the purposes of this study, a standardized difference of 0.1 indicated a small but meaningful difference between groups [26]. All analyses were conducted in SAS software, Version 9.4 (SAS Institute, Inc.).

## Results

### Physician representativeness

In total 47 physicians who completed the TRANSFORMATION survey were linked to health administrative databases and were compared to 69 physicians in the same practice groups, and 219 physicians practicing in the Eastern Ontario Public Health Unit. Participating physicians were similar in age. They included fewer female physicians when compared to the same practice group, but this was comparable to other physicians in eastern Ontario. TRANSFORMATION providers had roster sizes that were more similar to those of their colleagues in the same practice group and tended to be larger than those of other physicians working in the same geographical area. Physician survey participants also included more physicians who worked in community health centre (CHC) practices. They included fewer physicians working in fee-for-service models compared to the geographical area, with higher representation of physicians working in FHTs in the region (Table 2a).

**Table 2a:** Comparing physician participants of the TRANSFORMATION survey to other physicians in the same practice group and physicians in Eastern Ontario Public Health Unit (PHU)

	Group 1: Transformation physician respondents	Group 2: Physicians in same group of physician respondents	Group 3: Physicians in Eastern Ontario PHU	Standardized difference	
	N = 47	N = 69	N = 219	**Group 2 vs. 1**	**Group 3 vs. 1**
**Sex**, N (%)					
Female	14 (28.8)	29 (42.0)	67 (30.6)	0.26	0.02
Male	33 (70.2)	40 (58.0)	152 (69.4)	0.26	0.02
**Age**, Mean (SD)	53 (9)	52 (12)	53 (13)	0.09	0.09
**Years**, in practice, Mean (SD)	26 (10)	24 (13)	27 (13)	0.20	0.05
**Canadian medical graduate**, N (%)					
No	9 (19.2)	13 (18.8)	59 (26.9)	0.01	0.19
Yes	38 (80.9)	56 (81.2)	160 (73.1)	0.01	0.19
**Roster size**, Mean (SD)	1,657 (918)	1,509 (965)	885 (1015)	0.16	0.80
**Primary care model**, N (%)					
Group salaried with team (CHC)	6 (12.8)	15 (21.7)	15 (6.9)	0.24	0.20
Enhanced FFS (FHG&CCM&FFS)	13 (27.7)	6 (8.7)	147 (67.1)	0.51	0.86
Group capitated (FHN&FHO)	15 (31.9)	19 (28)	39 (17.8)	0.10	0.33
Group capitated with team (FHT)	13 (27.7)	29 (42.0)	18 (8.2)	0.31	0.52

SD, standard deviation; CHC, Community Health Centre, FHG, Family Health Group; CCM, Chronic Care Model; FFS, Fee for service (can include solo practice); FHN, Family Health Network; FHO, Family Health Organization; FHT, Family Health Team.

*Primary care models are classified according to type of practice model and remuneration: group salaried with team (CHC), enhanced fee for service (including family health groups, chronic care model, and fee for service), group capitated (Family Health Network and Family Health Organization), and group capitated with an allied health team (i.e. Family Health Team).

QUALICOPC physicians were compared to other physicians in their practices and in the province of Ontario in a previous study that was authored by the authors the current study [4] (Table 2b); this table was used with permission. Briefly, we found that physician respondents were, on average, younger, had fewer years of experience, and consisted of a higher proportion of physicians who attended medical school abroad [4]. Survey respondents also consisted of fewer solo physicians [4].

**Table 2b:** Comparing physician participants of the QUALICOPC study to other physicians in the practice group, and Ontario primary care physicians

	Group 1: QUALICOPC physician respondents	Group 2: QUALICOPC physicians’ practice groups	Group 3: Ontario primary care physicians	Standardized difference	
				Group 2 vs. 1	Group 3 vs. 1
	N = 175	N = 2,507	N = 9,758		
**Sex**, N (%)					
Female	98 (56.0)	1,177 (47.0)	4,110 (42.1)	0.18	0.28
Male	77 (44.0)	1,330 (53.0)	5,642 (57.8)	0.18	0.28
**Age**, mean (SD)	49 (10)	51 (11)	51 (12)	0.19	0.20
**Years in practice**, mean (SD)	23 (11)	25 (12)	25 (13)	0.20	0.21
**Canadian medical graduate**, N (%)					
Yes	141 (80.6)	1,878 (74.9)	7,054 (72.3)	0.14	0.20
No	34 (19.4)	629 (25.1)	2,698 (27.7)	0.14	0.20
**Roster size**, mean (SD)	1,257 (582)	1,126 (786)	1,120 (1,045)	0.19	0.16
Primary care model*, N (%)					
Solo physicians	12 (6.9)	0	3,711 (38.0)	-	0.81
Group enhanced FFS (FHG)	44 (25.1)	1,117 (44.6)	2,415 (24.8)	0.42	0.01
Group capitated (FHN)	< 6	27 (1.1)	202 (2.1)	-	-
Group capitated (FHO)	38 (21.7)	401 (16.0)	1,765 (18.1)	0.15	0.09
Group capitated with team (FHT)	73 (41.7)	923 (36.8)	1,594 (16.3)	0.10	0.58
Other group	< 6	39 (1.6)	71 (0.7)	-	-

SD, standard deviation; FHG, Family Health Group; FHN, Family Health Network; FHO, Family Health Organization; FHT, Family Health Team.

*Primary care models are classified according to type of practice model and remuneration: solo physicians (including enhanced fee for service and fee for service), group enhanced fee for service (i.e. Family Health Group), group capitated (i.e. Family Health Organization), and group capitated with an allied health team (i.e. Family Health Team). Family Health Network and Other group models were not included in the analysis as they each had fewer than 6 physician respondents in the QUALICOPC.

**Source**: Li A, Cronin S, Bai YQ, Walker K, Ammi M, Hogg W, et al. Assessing the representativeness of physician and patient respondents to a primary care survey using administrative data. BMC Fam Pract. 2018;19(77):1–10.

### Patient representativeness

534 patient participants of the TRANSFORMATION survey were compared to 1,523 patients who visited the same physicians on the day of survey administration, and 2,900 who visited the responding physicians on four randomly selected dates throughout the year. Compared to patients seeing physicians on the same day, survey participants consisted of a greater proportion of individuals between 45 and 64 years of age and fewer individuals who were 65 years of age or older. They consisted of fewer individuals with very high morbidity than others seeing the same physician on the same day and throughout the year. They were from more rural areas when compared to the same day, and to a lesser extent, throughout the year. Lastly, survey participants had fewer acute care visits and were less likely to have COPD or CHF compared to both same day and randomly selected day patients (Table 3a).

**Table 3a:** Comparing patient participants of the TRANSFORMATION survey to other patients visiting the practice on the same day and randomly selected dates

	Group 1: Transformation patient respondents	Group 2: Same Day Group	Group 3: Randomly Selected Day	Standardized difference	
	N = 534	N = 1523	N = 2,900	Group 2 vs. 1	Group 3 vs. 1
**Sex**, N (%)					
Female	325 (60.9)	903 (59.3)	1,690 (58.3)	0.03	0.05
Male	209 (39.1)	620 (40.7)	1,210 (41.7)	0.03	0.05
**Age**, N (%)					
18–44	138 (25.8)	357 (23.4)	852 (29.4)	0.06	0.08
45–64	244 (45.7)	516 (33.9)	934 (32.2)	0.24	0.28
>= 65	152 (28.5)	650 (42.7)	1,114 (38.4)	0.30	0.21
**Material deprivation** N (%)					
1 (lowest deprivation)	44 (8.4)	174 (12.0)	359 (13.2)	0.12	0.16
2	108 (20.6)	223 (15.4)	465 (17.1)	0.13	0.09
3	121 (23.0)	266 (18.4)	523 (19.2)	0.12	0.09
4	110 (20.95)	272 (18.8)	528 (19.4)	0.05	0.04
5 highest deprivation)	142 (27.1)	512 (35.4)	850 (31.2)	0.18	0.09
**Resource utilization bands (RUBs)**, N (%)					
0 (non-user)	17 (3.2)	12 (0.8)	51 (1.8)	0.17	0.09
1 (health user)	8 (1.5)	19 (1.3)	52 (1.8)	0.02	0.02
2 (low morbidity)	67 (12.6)	127 (8.3)	259 (8.9)	0.14	0.12
3 (moderate morbidity)	277 (51.9)	699 (45.9)	1,362 (47.0)	0.12	0.10
4 (high morbidity)	110 (20.6)	381 (25.0)	658 (22.7)	0.11	0.05
5 (very high morbidity)	55 (10.3)	285 (18.7)	518 (17.9)	0.24	0.22
**Rurality Index of Ontario**, N (%)					
Large urban	30 (5.6)	266 (17.5)	708 (24.4)	0.38	0.55
Small urban	184 (34.5)	767 (50.4)	1,080 (37.2)	0.33	0.06
Rural	320 (59.9)	490 (32.2)	1,112 (38.3)	0.58	0.44
**Health care visits in the last year**, Mean (SD)					
Primary care visits	3.66 (5.0)	4.06 (4.7)	4.30 (4.9)	0.08	0.13
ED visits	0.75 (1.53)	1.27 (2.5)	1.23 (2.5)	0.25	0.24
Acute care visits	0.15 (0.60)	0.31 (0.8)	0.30 (0.9)	0.21	0.20
**Chronic Disease**, N (%)					
Asthma	97 (18.2)	314 (20.6)	604 (20.8)	0.06	0.07
COPD	27 (5.1)	182 (12.0)	314 (10.8)	0.25	0.21
CHF	35 (6.6)	202 (13.3)	298 (10.3)	0.23	0.13
Hypertension	214 (40.1)	757 (49.7)	1,258 (43.4)	0.19	0.07
Diabetes	132 (24.7)	434 (28.5)	693 (23.9)	0.09	0.02

ED, emergency department; SD, standard deviation; COPD, chronic obstructive pulmonary disease; CHF, congestive heart failure.

A total of 1,225 patient participants of the QUALICOPC survey were linked to health administrative databases and compared to 29,885 patients who visited participating physicians on the same day, and 6,828 who visited the same physicians on four randomly selected dates throughout the year. Comparing the QUALICOPC group to those who visited on the same day, all standardized differences were under 0.1. QUALICOPC participants included a lower proportion of people with low morbidity compared to patients from randomly selected days throughout the year. In addition, there was a higher proportion of respondents with asthma in the QUALICOPC patient sample than in those attending on random days (Table 3b).

**Table 3b:** Comparing patient participants of the QUALICOPC survey to other patients visiting the practice on the same day and randomly selected dates

	Group 1: QUALICOPC patient respondents	Group 2: Same Day Group	Group 3: Randomly Selected Day Group	Standardized difference	
	N = 1,225	N = 29,885	N = 6,828	**Group 2 vs. 1**	**Group 3 vs. 1**
**Sex**, N (%)					
Female	782 (63.8)	18,429 (61.7)	3,806 (60.8)	0.04	0.06
Male	443 (36.2)	11,456 (38.3)	2,452 (39.2)	0.04	0.06
**Age**, N (%)					
18–44	423 (34.5)	10,955 (36.7)	2,311 (36.9)	0.04	0.05
45–64	492 (40.1)	11,083 (37.1)	2,246 (35.9)	0.06	0.09
>= 65	310 (25.3)	7,847 (26.3)	1,701 (27.18)	0.02	0.04
**Material deprivation**, N (%)					
1 (lowest deprivation)	303 (25.1)	5,333 (20.1)	1,108 (19.8)	0.02	0.02
2	266 (22.0)	5,417 (20.4)	1,147 (20.5)	0.05	0.05
3	208 (17.2)	5,224 (19.7)	1,061 (19.0)	0.02	0.00
4	219 (18.1)	5,085 (19.2)	1,144 (20.5)	0.00	0.03
5 highest deprivation)	211 (17.5)	5,474 (20.6)	1,133 (20.3)	0.05	0.04
**Resource utilization bands (RUBs)**, N (%)					
0 (non-user)	22 (1.8)	537 (1.8)	162 (2.6)	0.00	0.05
1 (health user)	35 (2.9)	716 (2.4)	171 (2.7)	0.03	0.01
2 (low morbidity)	87 (7.1)	2,914 (9.8)	651 (10.4)	0.096	0.12
3 (moderate morbidity)	662 (54.0)	15,514 (51.9)	3,342 (53.4)	0.04	0.01
4 (high morbidity)	292 (23.8)	7,026 (23.5)	1,330 (21.3)	0.01	0.06
5 (very high morbidity)	127 (10.4)	3,178 (10.6)	602 (9.6)	0.01	0.02
**Rurality Index of Ontario**, N (%)					
< 10	795 (64.9)	20,620 (69.0)	4,101 (65.53)	0.09	0.02
10 < = RIO < 40	306 (25.8)	7,094 (23.7)	1,546 (24.7)	0.05	0.03
40 +	114 (9.3)	2,171 (7.3)	611 (9.8)	0.07	0.02
**Healthcare visits in the last year**, Mean (SD)					
Primary care visits	5.83 (6.2)	6.11 (6.2)	5.33 (5.8)	0.05	0.08
ED visits	0.58 (1.2)	0.65 (1.6)	0.67 (1.8)	0.06	0.07
Acute care visits	0.12 (0.47)	0.14 (0.51)	0.14 (0.5)	0.03	0.03
**Chronic Disease**, N (%)					
Asthma	255 (20.8)	5,145 (17.2)	1,033 (16.5)	0.09	0.11
COPD	56 (4.6)	1,412 (4.7)	289 (4.6)	0.01	0.00
CHF	44 (3.6)	1,221 (4.1)	262 (4.2)	0.03	0.03
Hypertension	446 (36.4)	11,318 (37.9)	2,287 (36.6)	0.03	0.01
Diabetes	206 (16.8)	5,893 (19.7)	1,083 (17.3)	0.08	0.01

ED, emergency department; SD, standard deviation; COPD, chronic obstructive pulmonary disease; CHF, congestive heart failure.

## Discussion

This representativeness study compared patient respondents to other users of primary care across two primary care surveys that used different sampling and recruitment techniques. We found that patient respondents of the QUALICOPC survey were representative of other patients who accessed primary care on both the day of survey administration and throughout the year. However, patients responding to the TRANSFORMATION study comprised of more people between 45 and 64 years of age, were from rural areas, were lower users of the health care system and had lower incidence of chronic disease. We also found that physicians who responded to TRANSFORMATION differed compared to other physicians in the geographic area in terms of proportion of physicians trained in Canada and included more male physicians than physicians in their practices. QUALICOPC physicians were negligibly different from their practice groups but were found to be younger, included more females, and cared for larger rosters of patients than other physicians in the province [4].

The extent to which physicians differed from the larger intended population is similar to other literature emphasizing the different characteristics between physicians who volunteer for research studies, and those who do not [1, 3]. These studies suggest that physicians who participate are more likely to be female, as in our QUALICOPC physician findings, and work in team-based models of care [4, 28], which was noted in results of both TRANSFORMATION and QUALICOPC physician participants. Other studies have also suggested that it is difficult to recruit physicians working in fee for service models of primary care [28], resulting in the underrepresentation of this group in primary care surveys. This pattern of practices recruited was noted in physician respondents of the TRANSFORMATION survey, but not in the QUALICOPC respondent physicians, where the proportion of physicians working in fee for service models was similar to the proportion within the entire province. The TRANSFORMATION survey invested additional resources into physician recruitment and ensured a response rate of 41.3%, while the QUALICOPC survey sampled broadly but did not invest additional resources in physician recruitment and had a physician response rate of 3%.

The differences between the QUALICOPC and TRANSFORMATION patient representativeness was surprising as both studies used consecutive visit-based sampling, with the former using practice staff to distribute surveys while the latter used survey administrators. The visit-based method of recruitment has been reported to under-represent low users of care and not be representative of housebound patients [29]. In an Ontario study, a waiting room sample of patients was found to be older and more likely to be female than the overall practice population [28]. A similar finding was noted for QUALICOPC patient participants, where they were representative of patients visiting the practice, while TRANSFORMATION respondents were younger compared to other patients visiting their physicians. Similar to TRANSFORMATION, many surveys that recruit from clinic waiting rooms use a research assistant to either conduct all the recruitment, or to explain the study to participants and request informed consent after front desk staff have referred participants to them [7, 30]. In this study, survey distribution by practice staff (QUALICOPC) appeared to generate a more representative respondent sample than distribution by research staff (TRANSFORMATION). Further exploration of the difference between practice and research staff in this context is warranted, including the number of surveys distributed. In addition, the impact of survey recruitment on practice staff should be considered.

The QUALICOPC survey respondents included a higher proportion of individuals from the lowest quintile of material deprivation. The relationship between area deprivation and survey responses has been studied with a view to increasing response rates from these groups, with emphasis on using in person methods to facilitate responses [31]. Information about reasons for declining participation were not collected, thus, further investigation into the intersection of material deprivation and survey response rate is warranted.

### Limitations

While we used administrative datasets to derive physician and patient comparison groups, certain limitations must be noted. The variables used included health care utilization, health status, and sociodemographic characteristics. However, we did not include a measure of access to primary care, despite the fact that this is a key performance indicator for this sector, was measured as part of the QUALICOPC survey [3], and would have been relevant given the visit-based approach to sampling. In addition, we did not examine survey outcomes in relation to the characteristics of respondents [32]. Such an approach may have given a more accurate understanding of the effect of representativeness on results. We conducted this study in the province of Ontario, which has a single payer health system that includes physician primary care services; the findings may not be generalizable to jurisdictions where a different structure of primary care exists. Both surveys examined used in person, visit based sampling. Thus, our conclusions are not transferable to mail or email surveys, which have both been associated with different respondent populations in primary care [6, 33]. This limitation is particularly relevant given that the survey data analyzed are now several years old. The use of telehealth has increased over the past few years, and with it, the use of electronic surveys [34, 35]. Understanding representativeness of electronic surveys may help inform methods, and support interpretation of findings in context.

## Conclusion

This study contributes to a growing body of research on nonresponse bias in primary care surveys, with specific consideration to practice based sampling approaches. It is possible to recruit a convenience sample of patients in the waiting room who are representative of typical users of primary care. However, representativeness may be affected by the individual recruiting and distributing the surveys. With respect to physicians, targeted and selected recruitment resulted in a higher response rate for the TRANSFORMATION survey, but did not achieve a more representative sample of physicians than the less intensive and less expensive approach did. Our findings emphasize the need to measure and account for survey representativeness through techniques that can be applied during sampling, such as delayed sampling or oversampling [5, 9] of groups that are known to be poorly represented.

## Electronic supplementary material

Below is the link to the electronic supplementary material.

Supplementary File 1: A Brief Overview of Primary Care Models and Remuneration in Ontario

## Data Availability

The dataset from this study is held securely in coded form at ICES. While legal data sharing agreements between ICES and data providers (e.g., healthcare organizations and government) prohibit ICES from making the dataset publicly available, access may be granted to those who meet pre-specified criteria for confidential access, available at www.ices.on.ca/DAS (email: das@ices.on.ca). The full dataset creation plan and underlying analytic code are available from the authors ( Dr. Walter Wodchis, walter.wodchis@utoronto.ca) upon request, understanding that the computer programs may rely upon coding templates or macros that are unique to ICES and are therefore either inaccessible or may require modification.

## List of abbreviations

CAPE: Client Agency Program Enrolment
CHC: Community Health Centre
FFS: Fee for service
FHT: Family Health Team
OHIP: Ontario Health Insurance Plan
QUALICOPC: Quality and Costs of Primary Care Survey
RUB: Resource Utilization Band
