# Supplementary material for: How do respondents of primary care surveys compare to typical users of primary care? A comparison of two surveys
Source: BMC Prim Care. 2023 Mar 24;24:80. doi: 10.1186/s12875-023-02029-1 (PMC10037805; doi:10.1186/s12875-023-02029-1)
Supplement: Supplementary file 1 — A Brief Overview of Primary Care Models and Remuneration in Ontario [file 12875_2023_2029_MOESM1_ESM.pdf]

Supplementary File 1: A Brief Overview of Primary Care Models and Remuneration in Ontario

Primary Care Delivery Model	Composition and Characteristics	Physician Compensation Model	Patient enrolment required (Rostering*)
**Fee for Service (FFS)**	Solo physicians compensated for services performed according to the Schedule of Benefits.	Fee-for-service	No
**Comprehensive Care Model (CCM)**	Solo physicians providing comprehensive primary care services to enrolled patients and some after hours care.	Fee-for-service based plus after-hours premiums. Monthly capitation payments for enrolled patients.	Yes
**Family Health Group (FHG)**	Groups of physicians (3 or more) providing comprehensive care to enrolled patients on a 24/7 basis (through office hours and Telephone Health Advisory Services).	Fee for service plus after hours and comprehensive care premiums and bonuses, plus monthly comprehensive capitation payments.	Voluntary patient enrolment.
**Family Health Network (FHN)**	Groups of 3 or more physicians providing comprehensive care to patients through office hours and Telephone Health Advisory Service. **The main difference between FHN and FHO is the base rate payment and basket of core services.*	Capitated payments and fee-for-service.	Yes
**Family Health Organization (FHO)**	Groups of 3 or more physicians, providing care through regular and extended office hours, and nurse staffed Telephone Health Advisory Service. **The main differences between FHN and FHO are the base rate payment*	Blended capitation model, with additional payments for delivering targeted care services.	Yes

	and the basket of core services.		
**Family Health Team (FHT)**	Team of 3 or more physicians, with interdisciplinary health providers (nurses, social workers, dietitians, and others).	Physicians must be practicing within FHN or FHO, Blended capitation model, blended salary model, or complement-based remuneration plus bonuses and incentives.	Yes
**Community Health Centre (CHC)**	Team of physicians and interdisciplinary health providers. Health promotion and community programs also offered.	Salary	Yes, but also serves individuals who are not rostered
**Other Model**	Other group models, including Community Health Group, Group Health Centre, and Rural and Northern Physician Group	Various	Various

From: Li A, Cronin S, Bai YQ, Walker K, Ammi M, Hogg W, et al. Assessing the representativeness of physician and patient respondents to a primary care survey using administrative data. BMC Fam Pract. 2018;19(77):1–10

Sources: Marchildon GP, Hutchison B. Primary care in Ontario, Canada: New proposals after 15 years of reform. Health Policy (New York) [Internet]. 2016;120(7):732–8. Available from: http://dx.doi.org/10.1016/j.healthpol.2016.04.010

Rudoler D, Peckham A, Grudniewicz A, Marchildon G. Coordinating primary care services: A case of policy layering. Health Policy (New York) [Internet]. 2019;123(2):215–21. Available from: https://doi.org/10.1016/j.healthpol.2018.12.002
